# CaMKII orchestrates endoplasmic reticulum stress and apoptosis in doxorubicin‐induced cardiotoxicity by regulating the IRE1α/XBP1s pathway

**DOI:** 10.1111/jcmm.17560

**Published:** 2022-09-16

**Authors:** Lingheng Kong, Yimeng Zhang, Jiayi Ning, Chennian Xu, Zhenyi Wang, Jian Yang, Lifang Yang

**Affiliations:** ^1^ Department of Anaesthesiology Xi'an Children's Hospital Xi'an China; ^2^ Institute of Basic Medical Science Xi'an Medical University Xi'an China; ^3^ Department of Cardiovascular Surgery, Xijing Hospital Air Force Medical University Xi'an China; ^4^ Department of Cardiovascular Surgery General Hospital of Northern Theatre Command Shenyang China

**Keywords:** ADAMTS13‐TSP1 axis, apoptosis, CaMKII, cardiotoxicity, doxorubicin, endoplasmic reticulum stress, IRE1α/XBP1s pathway

## Abstract

Doxorubicin (Dox), an anthracycline antibiotic with potent antitumor effects, has limited clinical applications due to cumulative cardiotoxicity. Ca^2+^/calmodulin‐dependent protein kinase II (CaMKII) is implicated in the pathological progression of Dox‐induced cardiotoxicity. This study examined the hypothesis that CaMKII exacerbates Dox‐induced cardiotoxicity by promoting endoplasmic reticulum stress and apoptosis through regulation of the inositol‐requiring enzyme 1α (IRE1α)/spliced X‐box binding protein 1 (XBP1s) pathway. Our results demonstrated that CaMKII activation and IRE1α/XBP1s pathway were involved in Dox‐treated hearts. CaMKII inhibition with KN‐93 ameliorated Dox‐induced cardiac dysfunction and pathological myocardial changes. In addition, CaMKII inhibition prevented Dox‐induced endoplasmic reticulum stress and apoptosis. Moreover, CaMKII inhibition increased the expression of IRE1α and XBP1s in Dox‐treated hearts. The IRE1α inhibitor 4μ8C blocked the protective effect of CaMKII inhibition against Dox‐induced cardiotoxicity. Mechanistically, 4μ8C prevented the effects of CaMKII inhibition on Dox‐induced endoplasmic reticulum stress and apoptosis by inhibiting the expression of IRE1α and XBP1s. Additionally, treatment with rhADAMTS13 decreased the protein level of thrombospondin 1 (TSP1) and the phosphorylation of CaMKII in Dox‐treated human AC16 cardiomyocytes. Taken together, these results demonstrate that the ADAMTS13‐TSP1 axis regulates CaMKII activation and exacerbates Dox‐induced cardiotoxicity by triggering endoplasmic reticulum stress and apoptosis by inhibiting the IRE1α/XBP1s pathway.

## 1 INTRODUCTION

Despite improvements in early detection strategies and advances in therapeutics, cancer and cardiovascular diseases are the leading causes of morbidity and mortality worldwide.^1^, ^2^, ^3^ Anthracyclines, including doxorubicin (Dox), are widely used for the treatment of a variety of cancers.^4^ However, Dox‐induced time‐ and dose‐dependent cardiotoxicity is a serious side effect leading to left ventricular dysfunction or even heart failure.^5^ Although many studies have revealed that these outcomes may be related to reactive oxygen species accumulation, topoisomerase 2β, inflammation, Ca^2+^ handling abnormalities and autophagy dysfunction,^6^, ^7^ the pathophysiological mechanism of Dox‐induced cardiotoxicity remains to be fully elucidated.

In response to a broad range of cellular stresses, unfolded or misfolded proteins accumulate in the endoplasmic reticulum (ER) lumen, leading to unfolded protein response (UPR) activation and ER stress, as indicated by perturbations in intracellular Ca^2+^ homeostasis and protein synthesis and secretion, and this process has been implicated in a wide variety of cardiovascular diseases, including Dox‐induced cardiotoxicity.^8^, ^9^ The UPR is induced by three stress sensor proteins: inositol‐requiring enzyme 1α (IRE1α), PKR‐like ER kinase (PERK) and activating transcription factor 6 (ATF6).^9^ Under normal conditions, these factors are maintained in an inactive state by associating with glucose‐regulated protein 78 (GRP78).^8^ In response to ER stress, IRE1α is activated via oligomerization and splices X‐box binding protein 1 (XBP1) to form XBP1s. As a nuclear transcription factor, XBP1s upregulates the transcription of several genes involved in the UPR.^8^ The IRE1α/XBP1 pathway is a highly evolutionarily conserved signalling pathway for restoring ER homeostasis in response to ER stress and is also activated in the heart.^10^, ^11^ Some studies have shown that selective activation of the IRE1α/XBP1 pathway limits ER stress injury.^12^ However, a recent study indicated that the protein expression of IRE1α was increased in Dox‐induced cardiotoxicity.^13^ Therefore, the role of the IRE1α/XBP1 pathway in ER stress remains unclear.

Ca^2+^/calmodulin‐dependent protein kinase II (CaMKII) is a multifunctional serine/threonine kinase that mediates physiological and pathological responses in response to cardiac stresses.^14^, ^15^, ^16^ CaMKII is an important molecular integrator of intracellular Ca^2+^ homeostasis and redox signals.^17^, ^18^, ^19^, ^20^ Therefore, it is well accepted that chronic and persistent CaMKII activation has deleterious effects on cardiac function and structure. CaMKII has been associated with myocardial ischaemia reperfusion injury, cardiac hypertrophy, myocardial infarction and the development of heart failure.^21^, ^22^, ^23^, ^24^ There is evidence that in cardiomyocytes, the ADAMTS13‐thrombospondin 1 (TSP1) axis regulates CaMKII phosphorylation, which in turn is responsible for heart failure. TSP1 is highly expressed in large vessels and in the myocardium in diabetic animals, and it has been shown to bind ADAMTS13 to form protein complexes.^25^, ^26^ However, the cellular mechanisms through which CaMKII regulates cardiac structure and function in Dox‐induced cardiotoxicity are less clear. In this study, we aimed to investigate whether ADAMTS13‐TSP1 could regulate CaMKII activation and whether CaMKII inhibition improved Dox‐induced cardiotoxicity by inhibiting ER stress by regulating the IRE1α/XBP1 pathway.

## 2 MATERIALS AND METHODS

### 2.1 Reagents

Antibodies against CaMKII, p‐CaMKII, phospholamban (PLB), IREα, XBP1s, PERK, GRP78, TSP1 and GAPDH were purchased from Cell Signalling Technology. The antibody against p‐PLB was purchased from Badrilla (Leeds). Antibodies against caspase‐3, cleaved caspase‐3 and ADAMTS13 were purchased from ABclonal Technology. The Pierce BCA protein assay kit was purchased from Thermo Scientific. KN‐93 was purchased from Tocris Bioscience. 4μ8C was purchased from Selleck (Houston). Recombinant human (rh) ADAMTS13 was purchased from R&D Systems. Doxorubicin (Dox) and dimethylsulfoxide (DMSO) were purchased from Sigma. The LDH enzyme‐linked immunoassay (ELISA) kit and lysis buffer were purchased from Beyotime Biotechnology. The terminal deoxynucleotidyl transferase dUTP nick end labelling (TUNEL) kit and protease and phosphatase inhibitor cocktails were purchased from Roche. Goat anti‐rabbit and goat anti‐mouse secondary antibodies were purchased from Zhongshan Company.

### 2.2 Animals

Adult male C57BL/6J mice weighing 20–25 g were purchased from the Laboratory Animal Centre of Xi'an Jiao Tong University (Xi'an, China). All experimental protocols for this study were approved by the Ethics Committee of Xi'an Jiao Tong University. The animals were housed in a controlled environment (12‐h light/dark cycle; 22–25°C; 55%–60% humidity) with free access to mouse chow and water.

### 2.3 Experimental protocol

**FIGURE 1:**
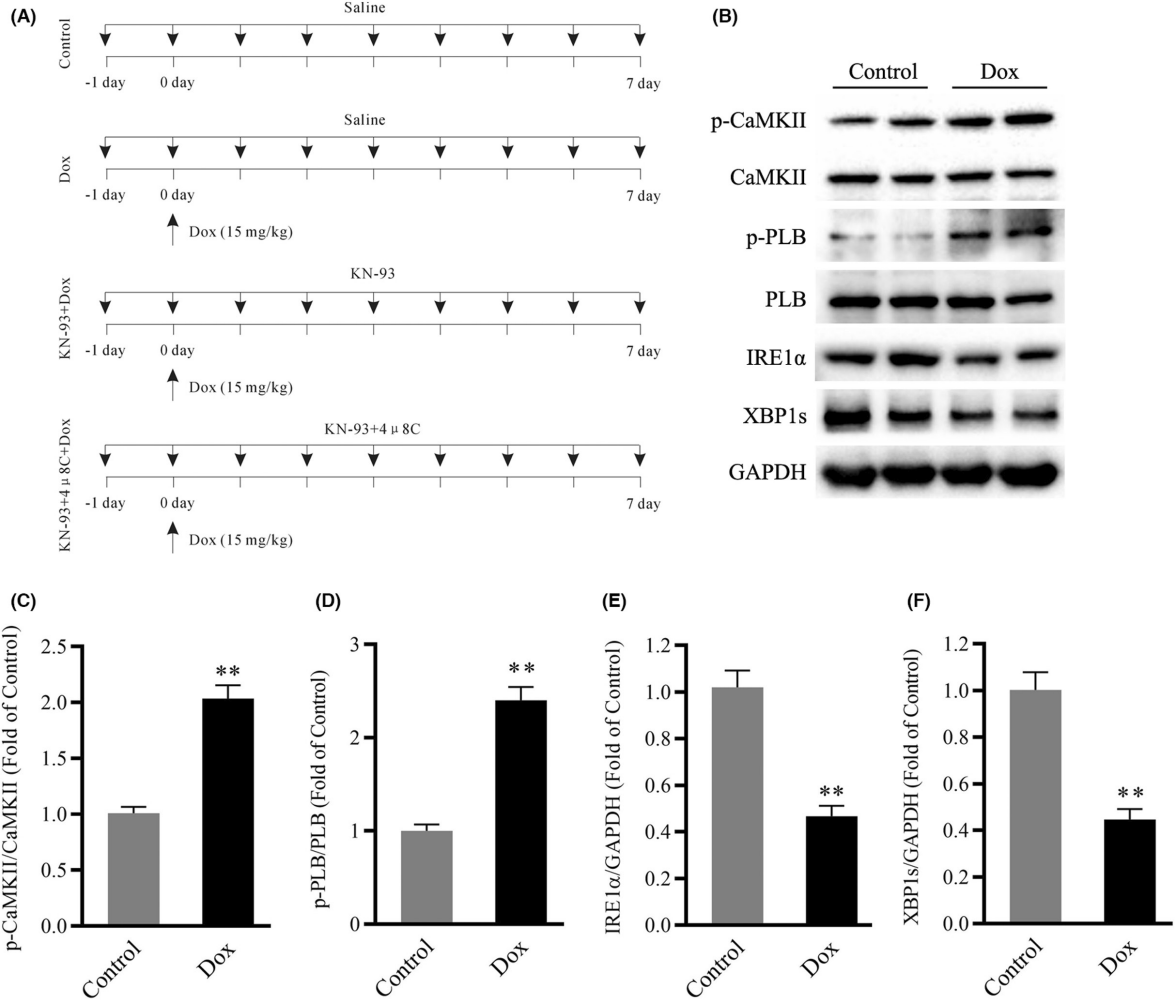
CaMKII activity and the expression of IRE1α/XBP1s in Dox‐treated mouse hearts. (A) Schematic representation of the experimental procedure. (B) Representative immunoblots showing p‐CaMKII, CaMKII, p‐PLB, PLB, IRE1α, XBP1s and GAPDH (internal control). (C–F) Statistical analysis of p‐CaMKII/CaMKII, p‐PLB/PLB, IRE1α and XBP1s, each protein was normalized to GAPDH. The data are presented as the mean ± SEM, *n* = 6. **p < 0.01 versus Control.

C57BL/6J mice were used for this study and were randomly divided into different groups (*n* = 15 each) as follows (Figure 1A): the control group (control), doxorubicin group (Dox), Dox plus KN‐93 group (KN‐93 + Dox) and Dox plus KN‐93 and 4μ8C group (KN‐93 + 4μ8C + Dox) group. The mice in the Dox, KN‐93 + Dox and KN‐93 + 4μ8C + Dox groups were intraperitoneally injected with 15 mg/kg Dox for one time. KN‐93 (5 mg/kg), 4μ8C (10 mg/kg) or the same volume of saline was intraperitoneally injected every day for a total of 8 times (1 day before the initial Dox treatment). Dox, KN‐93 and 4μ8C were dissolved in DMSO and diluted to a final concentration of 0.1% DMSO with normal saline. The doses of the drugs used in this study were based on previous studies.^27^, ^28^, ^29^ All mice in each group were monitored daily to observe their survival. The number of deaths was recorded at the same time point each day.

### 2.4 Echocardiography and haemodynamic measurement

C57BL/6J mice were anaesthetized with 2% isoflurane 7 days after the injection of Dox. The hearts were measured in the short‐axis by two‐dimensional and M‐mode using a Vevo 2100 ultrasound imaging system (VisualSonics). Images were used to analyse the parameters of cardiac function, including ejection fraction (EF), fractional shortening (FS), heart rate, left ventricular internal diameter at end‐diastole (LVIDd) and left ventricular posterior wall thickness (LVPWd), with Vevo software.

### 2.5 Histopathological examination

The hearts were washed with precooled PBS and fixed in 4% paraformaldehyde at room temperature for 48 h. Then, the hearts were embedded with paraffin for histology. Haematoxylin and eosin (HE) and Masson's trichrome staining were used to evaluate heart morphology and the degree of myocardial fibrosis, respectively.

### 2.6 Transmission electron microscopy

Ultrastructural examination of the ER was performed using transmission electron microscopy. The mouse heart tissues were cut into 2 mm cubes and immersed in 2% glutaraldehyde at 4°C for 24 h. Then, the tissue samples were cut into 70 nm‐thick sections with an ultramicrotome. The sections were stained with 1% uranyl acetate and 2% lead citrate and observed using a transmission electron microscope (HT7800; Hitachi). The ER luminal width was calculated using Image J software.

### 2.7 TUNEL staining

The TUNEL staining method was used to measure the apoptotic rate using an in situ cell death detection kit according to the manufacturer's instructions. Images were observed using an Olympus FV1000 confocal microscope (Olympus). The TUNEL‐positive myocytes showed green nuclear staining (green) and were counted in five randomly selected fields under high‐power magnification. The apoptotic rate was determined by calculating the ratio between the number of positive cells and the total number of myocytes and multiplying that number by 100%.

### 2.8 Determination of LDH activity by ELISA

The activity of serum LDH was measured using commercial ELISA kits from Beyotime Biotechnology in accordance with the manufacturer's instructions.

### 2.9 Cell culture model

Human AC16 cardiomyocytes were purchased from the Cell Bank of the Chinese Academy of Sciences and were cultured in Dulbecco's modified Eagle's medium (DMEM; HyClone) supplemented with 10% foetal bovine serum (Gibco), penicillin (100 IU/ml) and streptomycin (100 μg/ml) in a humid atmosphere with 5% CO_2_ and 95% air at 37°C. AC16 cardiomyocytes were treated with Dox (1 μmol/L) to mimic cardiotoxicity and then treated with rhADAMTS13 (0.3 μg/ml) to determine its effects on Dox‐induced CaMKII activation.

### 2.10 Western blotting

Proteins were extracted from the heart tissue using lysis buffer containing 1% protease and phosphatase inhibitor cocktails. A BCA assay kit (Thermo Fisher Scientific) was used to determine the protein concentrations. Equal amounts of proteins (40 μg) were separated by SDS–PAGE (10% or 12%) and transferred to PVDF membranes. The membranes were blocked using 5% (w/v) nonfat skim milk for 2 h at room temperature, followed by incubation with the primary antibodies at 4°C overnight. Subsequently, horseradish peroxidase (HRP)‐conjugated secondary antibodies were added and incubated with the PVDF membranes for 2 h at room temperature. Enhanced chemiluminescence reagents were used to detect the antigen–antibody complexes with a ChemiDoc XRS (Bio‐Rad). GAPDH was used as the internal control.

### 2.11 Statistical analysis

All of the statistical tests were performed in GraphPad Prism software (Version 8.0). The data are presented as the mean ± standard error of the mean (S.E.M.). Statistical analysis was performed by using one‐way analysis of variance (anova), followed by Tukey's post‐hoc tests, and *p* values <0.05/0.01 were considered to be statistically significant.

## 3 RESULTS

### 3.1 Dox treatment is associated with CaMKII activation and the downregulation of IRE1α and XBP1s expression

First, we assessed CaMKII activation and the protein levels of IRE1α and XBP1s in mouse hearts in the control and Dox groups. Our results showed CaMKII activation in the Dox group compared with the control group, as indicated by the phosphorylation of CaMKII at Thr286 and the phosphorylation of PLB at Thr17 (Figure 1B–D). In addition, compared with those in the control group, the Western blot results indicated that the protein levels of IRE1α and XBP1s were significantly downregulated in the Dox group (Figure 1B,E and F). These results indicated that CaMKII activation and the IRE1α/XBP1s pathway were involved in Dox‐induced cardiotoxicity.

### 3.2 CaMKII inhibition prevented Dox‐induced cardiac dysfunction and pathological myocardial changes

**FIGURE 2:**
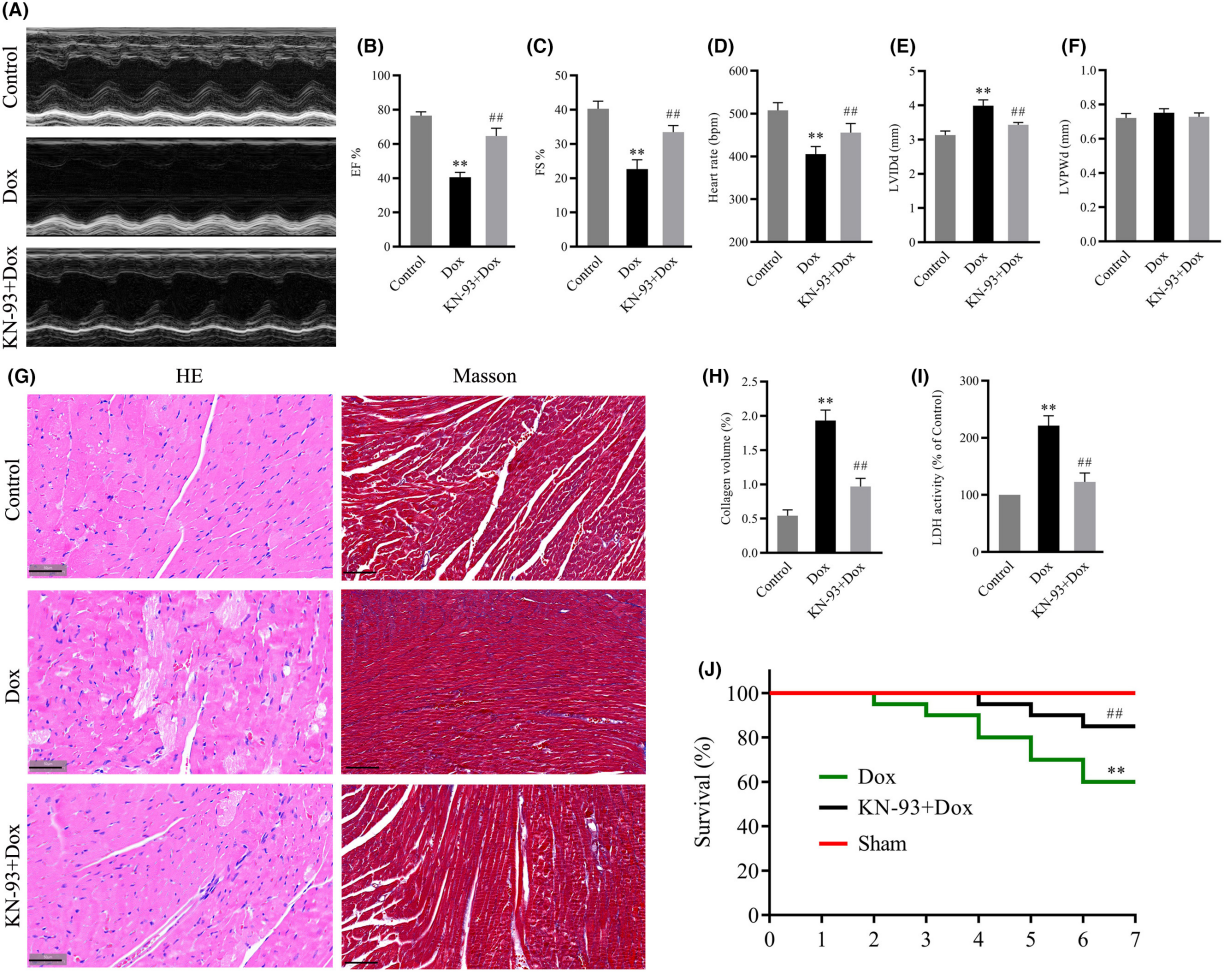
Effects of CaMKII inhibition with KN‐93 on cardiac function and structure in Dox‐treated mouse hearts. (A) Representative images of echocardiograms of hearts. (B–F) Group results of EF, FS, heart rate, LVIDd and LVPWd. (G) The left panel shows images with HE staining; the right panel shows images with Masson's staining (Scale bar: 50 μm). (H) Quantitative analysis of collagen volume. (I) LDH activity. (J) Survival curves. The data are presented as the mean ± SEM, *n* = 6. **p < 0.01 versus Control, ^##^ *p* < 0.01 versus Dox.

To investigate the effects of CaMKII in Dox‐induced cardiotoxicity, KN‐93, a potent and selective inhibitor of CaMKII, was used. Cardiac function and pathological myocardial changes were determined after treatment with Dox for 7 days. Compared with those in the control group, cardiac function parameters, including EF, FS and heart rate, were notably decreased, while LVIDd but not LVPWd was increased in the Dox group (Figure 2A–F). Serum LDH activity was significantly increased in Dox‐treated mice (Figure 2I). In addition, HE staining and Masson's trichrome staining showed that the myofibrillar component was disorganized and degenerated with deposition of fibrotic tissue in Dox‐treated hearts (Figure 2G,H). In addition, survival was significantly decreased in the Dox‐treated group (Figure 2J). As expected, CaMKII inhibition with KN‐93 alleviated cardiac dysfunction, as confirmed by the increases in EF, FS and heart rate, the decrease in LVIDd, improved histopathological changes and survival and attenuated serum LDH activity (Figure 2A–J). These results confirmed that CaMKII inhibition could prevent Dox‐induced cardiotoxicity.

### 3.3 CaMKII inhibition protected against Dox‐induced ER stress and apoptosis

**FIGURE 3:**
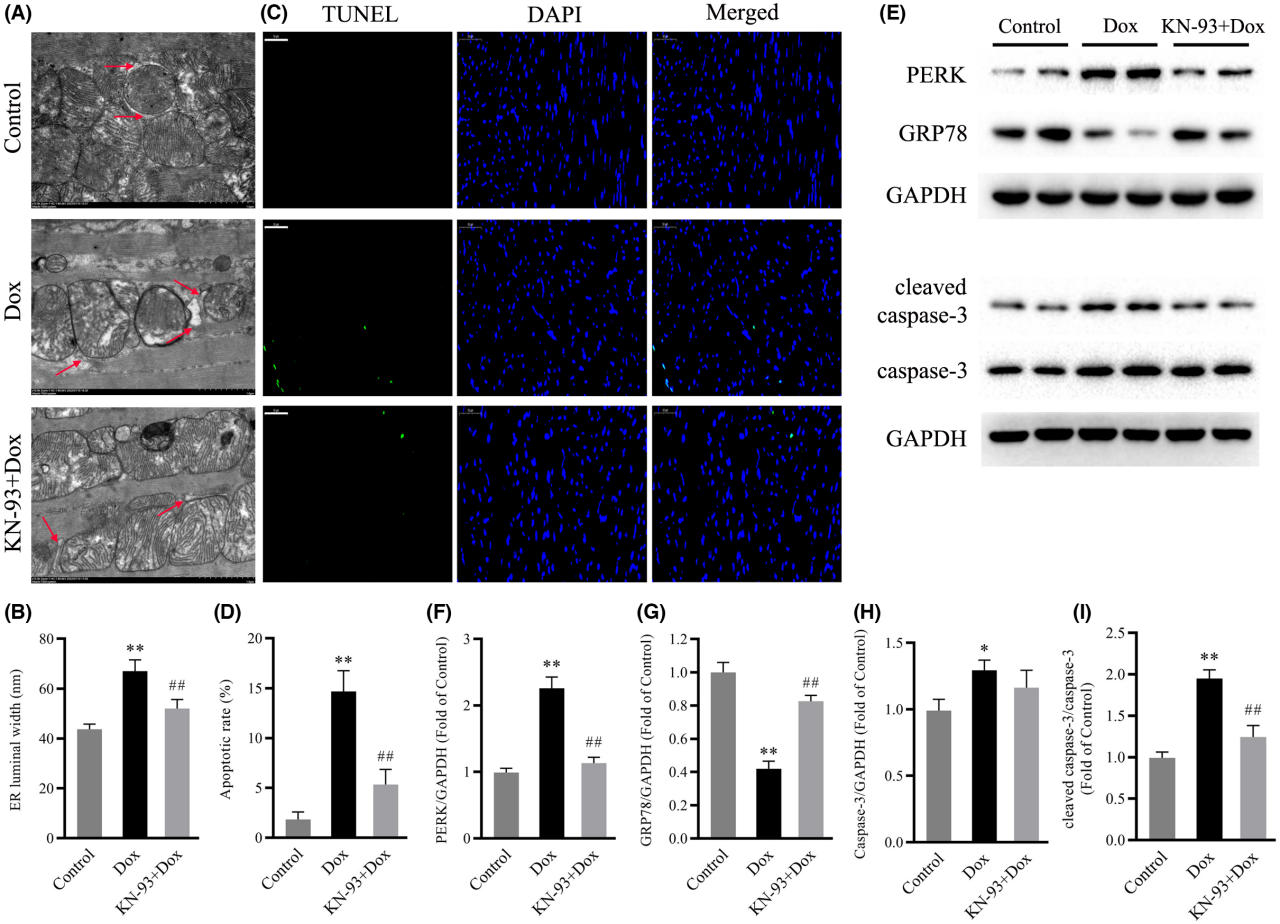
Effects of CaMKII inhibition with KN‐93 on ER stress and apoptosis in Dox‐treated mouse hearts. (A) Representative electron microscope images of the ER in the different groups (magnification: 15,000×). Arrows indicate ER. (B) Quantification of ER luminal width. (C) Representative TUNEL staining images in the different groups (Scale bar: 50 μm). (D) Apoptotic rate. (E) Representative immunoblots of PERK, GRP78, caspase‐3, cleaved caspase‐3 and GAPDH (internal control). (F–I) Statistical analysis of PERK, GRP78, caspase‐3, cleaved caspase‐3 and cleaved caspase‐3/caspase‐3; each protein was normalized to GAPDH. The data are presented as the mean ± SEM, *n* = 6. **p < 0.01 versus Control, ^##^ *p* < 0.01 versus Dox.

Some studies have revealed that endoplasmic reticulum stress and apoptosis play important roles in the irreversible cardiac remodelling associated with Dox‐induced cardiotoxicity.^30^, ^31^ Consistent with previous studies, our results confirmed that Dox treatment resulted in marked ER stress and apoptosis in mouse hearts, as indicated by increased ER dilation, the protein levels of PERK, caspase‐3 and cleaved caspase‐3, the ratio of cleaved caspase‐3/caspase‐3 and TUNEL‐positive cells, and decreased protein levels of GRP78 (Figure 3A–I). Interestingly, CaMKII inhibition with KN‐93 decreased ER dilation, the protein levels of PERK, caspase‐3 and cleaved caspase‐3, the ratio of cleaved caspase‐3/caspase‐3 and TUNEL‐positive cells and increased the protein level of GRP78 (Figure 3A–I). These data demonstrated that CaMKII inhibition protected against Dox‐induced ER stress and apoptosis in mouse hearts.

### 3.4 CaMKII inhibition increased Dox‐induced IRE1α and XBP1s downregulation

**FIGURE 4:**
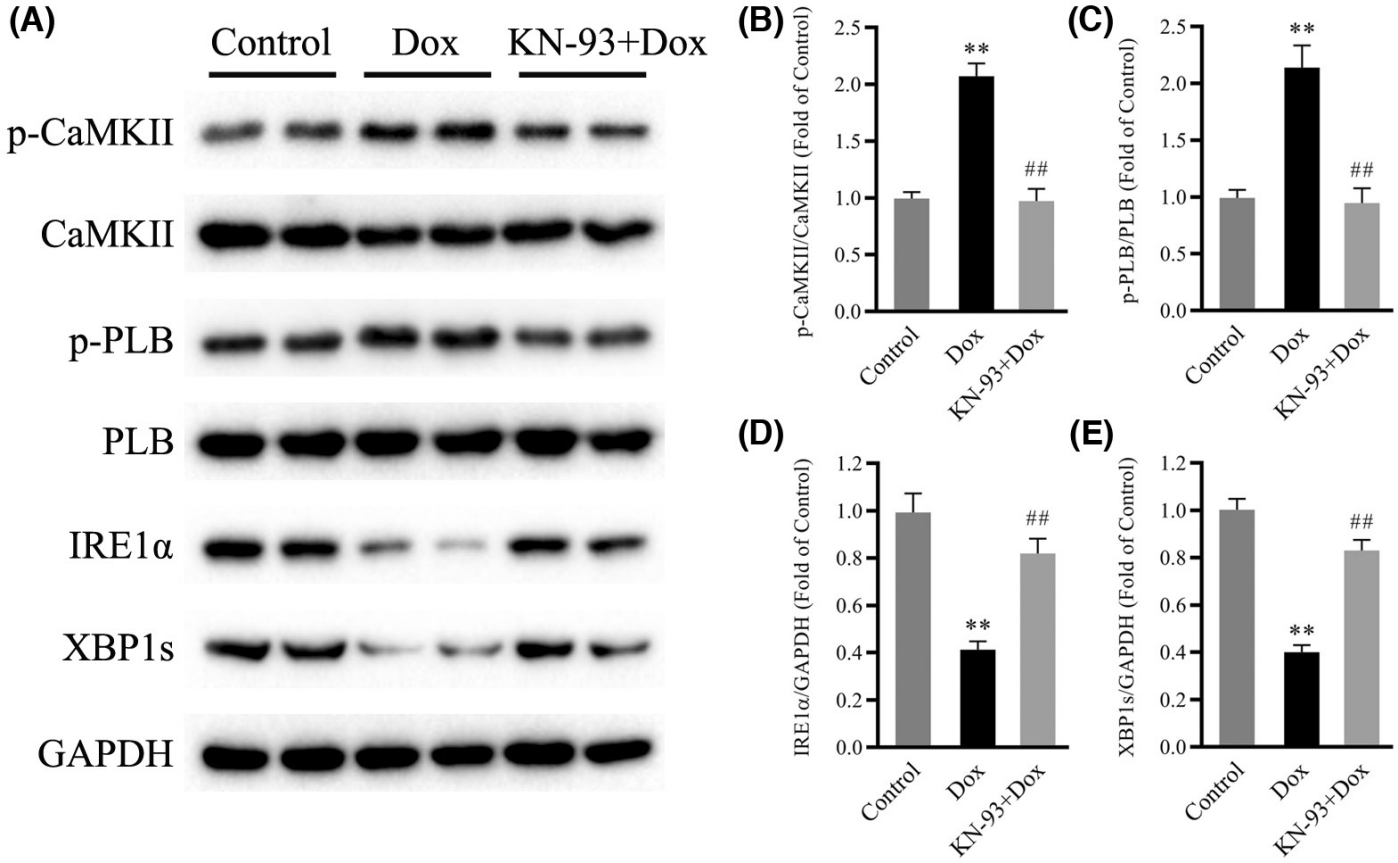
Effects of CaMKII inhibition with KN‐93 on IRE1α/XBP1s in Dox‐treated mouse hearts. (A) Representative immunoblots of p‐CaMKII, CaMKII, p‐PLB, PLB, IRE1α, XBP1s and GAPDH (internal control). (B–E) Statistical analysis of p‐CaMKII/CaMKII, p‐PLB/PLB, IRE1α and XBP1s. each protein was normalized to GAPDH. The data are presented as the mean ± SEM, *n* = 6. **p < 0.01 versus Control, ^##^ *p* < 0.01 versus Dox.

To determine whether IRE1α/XBP1s signalling contributes to the protective effects of CaMKII inhibition against Dox‐induced cardiotoxicity, we measured the expression of IRE1α and XBP1s in mouse hearts. Consistent with the previous findings, Western blot analysis showed robust downregulation of the protein levels of IRE1α and XBP1s in Dox‐treated hearts but significantly increased levels of CaMKII and PLB phosphorylation (Figure 4A–E). Notably, treatment with KN‐93 profoundly upregulated the protein levels of IRE1α and XBP1s in Dox‐treated hearts and reduced the phosphorylation of CaMKII and PLB (Figure 4A–E). These results indicated that IRE1α/XBP1s signalling was involved in the protective effects of CaMKII inhibition against Dox‐induced cardiotoxicity.

### 3.5 4μ8C‐mediated blocked the protective effects of CaMKII inhibition on Dox‐induced cardiac dysfunction and pathological myocardial changes

**FIGURE 5:**
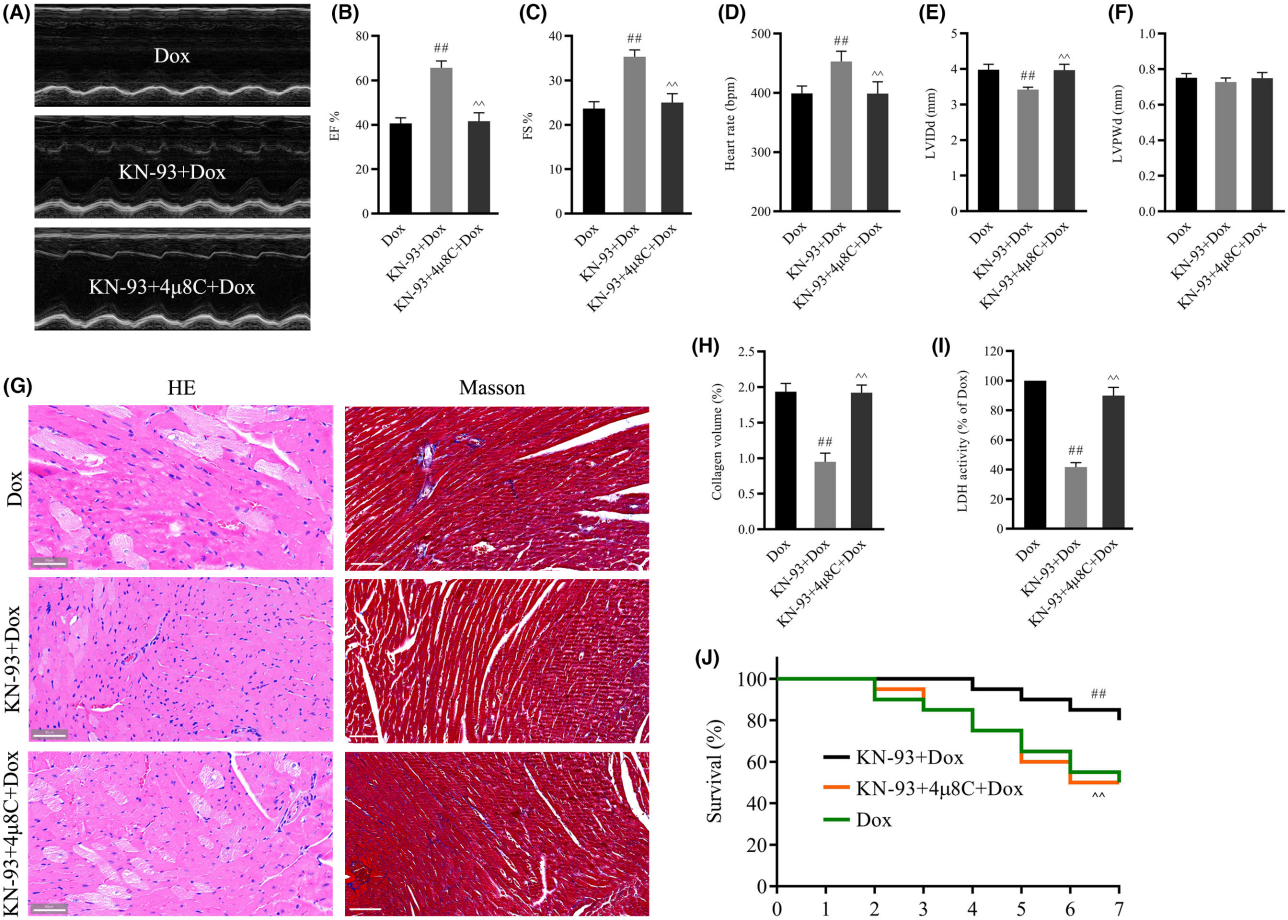
4μ8C inhibited the effects of CaMKII inhibition on cardiac function and structure in Dox‐treated mouse hearts. (A) Representative images of echocardiograms of the hearts. (B–F) EF, FS, heart rate, LVIDd and LVPWd. (G) The left panel shows images with HE staining. The right panel shows images with Masson's staining (Scale bar: 50 μm). (H) Quantitative analysis of collagen volume. (I) LDH activity. (J) Survival curves. The data are presented as the mean ± SEM, *n* = 6. ^##^ *p* < 0.01 versus Dox, ^^^^ *p* < 0.01 versus KN‐93 + Dox.

To determine whether IRE1α/XBP1s signalling contributes to the protective effects of CaMKII inhibition against Dox‐induced cardiotoxicity, the mice were administered an intraperitoneal injection of 4μ8C before KN‐93 treatment for 7 days. Compared with that in the Dox group, KN‐93 alleviated Dox‐induced cardiotoxicity, as confirmed by elevated EF, FS, and heart rate, decreased LVIDd, improved histopathological changes and survival and attenuated serum LDH activity (Figure 5A–J). More importantly, the protective effects of CaMKII inhibition against Dox‐induced cardiotoxicity were blocked by 4μ8C (Figure 5A–J). These results suggested that CaMKII inhibition protected against Dox‐induced cardiotoxicity by activating IRE1α/XBP1s signalling.

### 3.6 4μ8C prevented the effects of CaMKII inhibition on ER stress and apoptosis in Dox‐treated hearts

**FIGURE 6:**
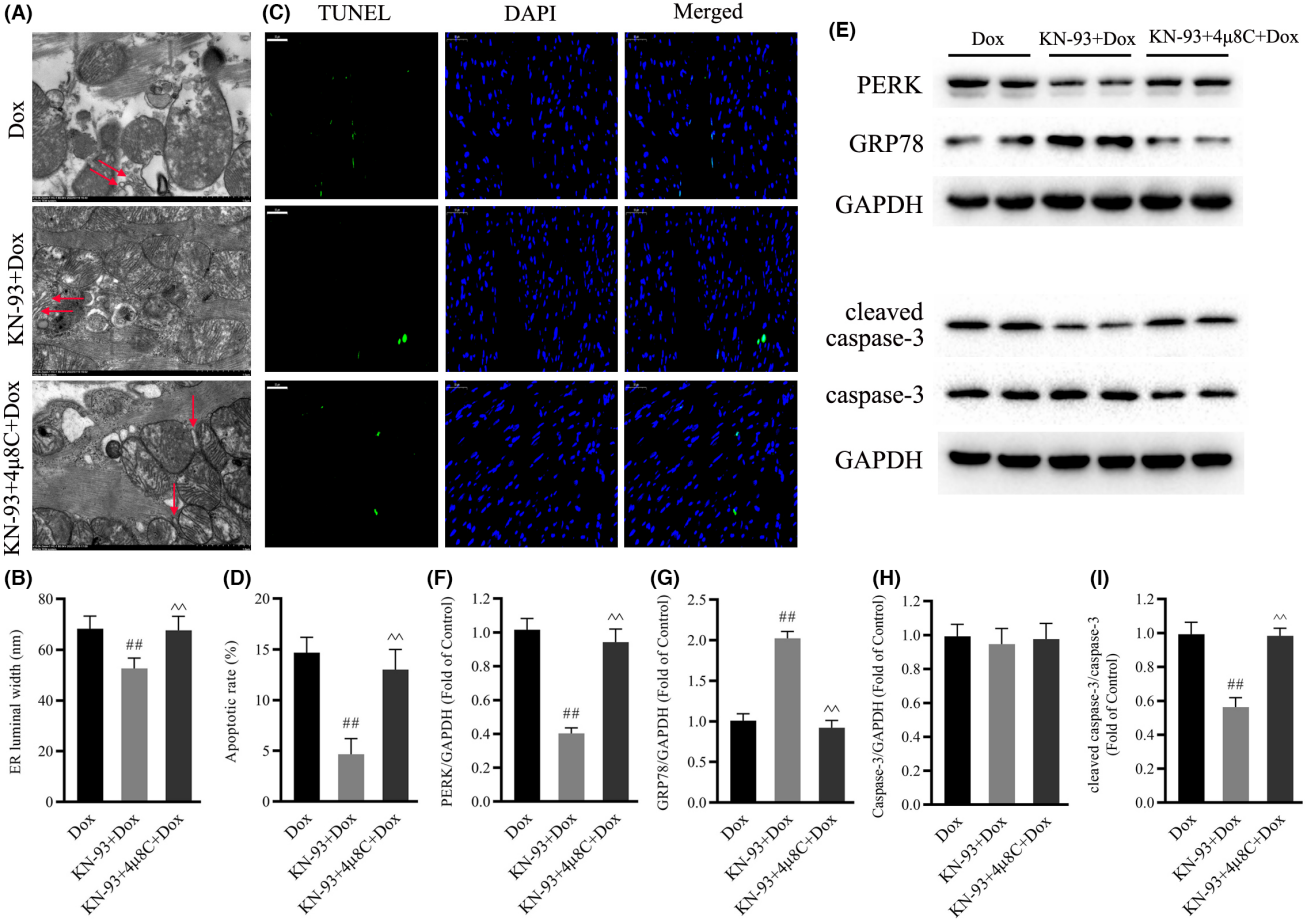
4μ8C prevented the effects of CaMKII inhibition on ER stress and apoptosis in Dox‐treated mouse hearts. (A) Representative electron microscope images of the ER in the different groups (magnification: 15000×). Arrows indicate ER. (B) Quantification of ER luminal width. (C) Representative TUNEL staining images in the different groups (Scale bar: 50 μm). (D) Apoptotic rate. (E) Representative immunoblots of PERK, GRP78, caspase‐3, cleaved caspase‐3 and GAPDH (internal control). (F–I) Statistical analysis of PERK, GRP78, caspase‐3, cleaved caspase‐3 and cleaved caspase‐3/caspase‐3; each protein was normalized to GAPDH. The data are presented as the mean ± SEM, *n* = 6. ^##^ *p* < 0.01 versus Dox. ^^^^ *p* < 0.01 versus KN‐93 + Dox.

Next, we investigated whether IRE1α/XBP1s signalling was involved in the effects of CaMKII inhibition on ER stress and apoptosis in Dox‐treated hearts. Compared with those in the Dox group, KN‐93 decreased ER dilation, the protein levels of PERK and cleaved caspase‐3, the ratio of cleaved caspase‐3/caspase‐3 and TUNEL‐positive cells and increased the protein level of GRP78 in Dox‐treated hearts (Figure 6A–I). Strikingly, 4μ8C abrogated the effects of KN‐93 on the protein levels of PERK, cleaved caspase‐3 and GRP78, ER dilation and TUNEL‐positive cells in Dox‐treated hearts (Figure 6A–I). These results suggested that the effects of CaMKII inhibition on ER stress and apoptosis in Dox‐treated hearts were related to IRE1α/XBP1s signalling.

### 3.7 4μ8C abolished the effect of CaMKII inhibition on Dox‐induced IRE1α and XBP1s downregulation

**FIGURE 7:**
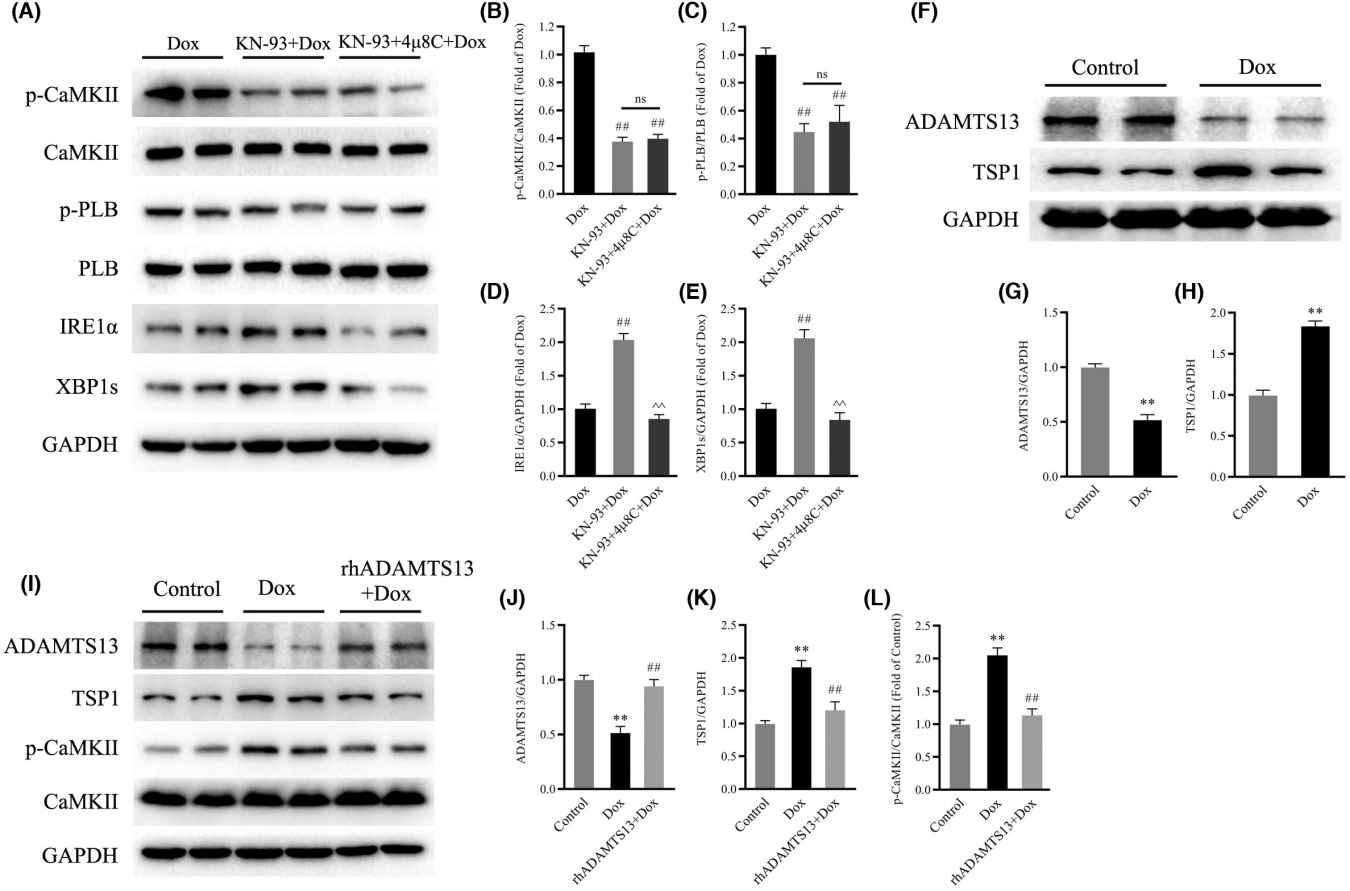
4μ8C blocked the effects of CaMKII inhibition on IRE1α/XBP1s in Dox‐treated mouse hearts and rhADAMTS13 affects CaMKII activation in Dox‐treated AC16 cardiomyocytes. (A) Representative immunoblots of p‐CaMKII, CaMKII, p‐PLB, PLB, IRE1α, XBP1s and GAPDH (internal control). (B–E) Statistical analysis of p‐CaMKII/CaMKII, p‐PLB/PLB, IRE1α and XBP1s. Each protein was normalized to GAPDH. The data are presented as the mean ± SEM, *n* = 6. ^##^ *p* < 0.01 versus Dox, ^^^^ *p* < 0.01 versus KN‐93 + Dox. (F) Representative immunoblots of ADAMTS13, TSP1 and GAPDH (internal control). (G and H) Statistical analysis of ADAMTS13 and TSP1. Each protein was normalized to GAPDH. (I) Representative immunoblots of ADAMTS13, TSP1, p‐CaMKII, CaMKII and GAPDH (internal control). (J–L) Statistical analysis of ADAMTS13, TSP1 and p‐CaMKII/CaMKII. Each protein was normalized to GAPDH. The data are presented as the mean ± SEM, *n* = 6. **p < 0.01 versus Control, ^##^ *p* < 0.01 versus Dox.

We further examined the mechanisms that revealed the role of IRE1α/XBP1s signalling in the effect of CaMKII inhibition on Dox‐treated mouse hearts. Consistent with the results shown in Figure 5, Western blot analysis showed that the protein levels of IRE1α and XBP1s were significantly downregulated in Dox‐treated hearts, but the levels of phosphorylation of CaMKII and phosphorylation of PLB were significantly elevated (Figure 7A–E). Moreover, treatment with KN‐93 profoundly upregulated the protein levels of IRE1α and XBP1s in Dox‐treated hearts and reduced the phosphorylation of CaMKII and PLB (Figure 7A–E). However, 4μ8C significantly reduced the protein levels of IRE1α and XBP1s induced by CaMKII inhibition in Dox‐treated hearts but had little effect on the levels of CaMKII phosphorylation or PLB phosphorylation (Figure 7A–E). Interestingly, compared with those in the Dox group, treatment with 4μ8C had little effect on the phosphorylation of CaMKII and PLB and the protein levels of IRE1α and XBP1s in Dox‐treated hearts (Figure S1A–E).

### 3.8 The ADAMTS13‐TSP1 axis regulated CaMKII activation

Given that the ADAMTS13‐TSP1 axis regulates CaMKII activation, which in turn is responsible for heart failure, we measured the expression of ADAMTS13 and TSP1 in mouse hearts. Western blot analysis showed that, compared with those in the control group, the protein level of ADAMTS13 was decreased and the protein level of TSP1 was increased in Dox‐treated mouse hearts (Figure 7F–H). To investigate whether the ADAMTS13‐TSP1 axis affects CaMKII activation in Dox‐induced cardiotoxicity, human AC16 cardiomyocytes were exposed to Dox and treated with rhADAMTS13. Consistent with the results obtained in vivo, Western blot analysis showed that the protein level of ADAMTS13 was decreased and the protein level of TSP1 was increased in Dox‐treated human AC16 cardiomyocytes compared with those in the control group (Figure 7I–K). In addition, we found that the phosphorylation of CaMKII was also increased (Figure 7I,L). Importantly, treatment with rhADAMTS13 increased the protein level of ADAMTS13, although the protein level of TSP1 and the phosphorylation of CaMKII were decreased in Dox‐treated human AC16 cardiomyocytes (Figure 7I–L).

## 4 DISCUSSION

Despite advances in cancer treatment strategies, including conventional chemotherapy and targeted therapies, which have led to improvements in cancer patient survival, treatment is associated with an increased risk of cardiotoxicity, such as myocyte destruction, left ventricular dysfunction and heart failure.^32^, ^33^ The incidence of cancer treatment‐induced cardiovascular injury has been widely noted, and cardiovascular disease is the predominant cause of mortality in cancer patients.^1^ However, the pathological mechanisms of Dox‐induced cardiotoxicity are complex and have not been fully elucidated. It has been suggested that reactive oxygen species and Ca^2+^ play pivotal roles in disturbing cardiomyocyte homeostasis in Dox‐induced cardiotoxicity.^34^, ^35^ In addition, evidence shows that diverse pathological conditions can induce the accumulation of unfolded or misfolded proteins within the ER and induce ER stress.^36^, ^37^, ^38^ In this study, our results indicated CaMKII activation and the IRE1α/XBP1s pathway inhibition in Dox‐treated mouse hearts and demonstrated the beneficial effects of CaMKII inhibition on reducing ER stress and apoptosis through the IRE1α/XBP1s pathway in mouse hearts with Dox‐induced cardiotoxicity. In addition, we found that the ADAMTS13‐TSP1 axis regulated CaMKII activation in Dox‐treated human AC16 cardiomyocytes. These findings provide a novel mechanism for Dox‐induced cardiotoxicity, which suggests that CaMKII is a potential target for the prevention of Dox‐induced cardiotoxicity.

It is well known that anthracyclines, such as Dox, can induce permanent cardiomyocyte injury, which is associated with increased cardiac fibrosis, cell apoptosis and left ventricular dysfunction, leading to irreversible cardiac remodelling and heart failure.^5^ The evidence shows that left ventricular dysfunction and cardiac fibrosis have crucial roles in the pathogenesis of Dox‐induced cardiotoxicity.^39^, ^40^ In addition, some studies have suggested that CaMKII activation is an important pathogenic factor in ischaemia‐ and Dox‐induced necroptotic cell death.^29^ Consistent with previous studies, our results further confirmed that Dox‐induced pathological structural damage and cardiac dysfunction, as indicated by HE and Masson's staining, decreased EF, FS and heart rate, and increased LVIDd and LDH activity. KN‐93, a selective inhibitor of CaMKII, improved Dox‐induced pathological structural damage and cardiac dysfunction, demonstrating that CaMKII inhibition could play a protective role in Dox‐induced cardiotoxicity.

Endoplasmic reticulum stress and apoptosis are key pathogenic factors in Dox‐induced cardiotoxicity.^41^, ^42^ Undoubtedly, perturbations in proteostasis result in the abnormal accumulation of unfolded or misfolded proteins in the ER, which is a condition referred to as ER stress.^8^ Over the past decade, a growing body of evidence has indicated pivotal roles of ER stress in the pathogenesis and development of cardiovascular diseases.^43^ In addition, numerous studies have shown that ER stress is associated with extreme levels of apoptosis in cardiovascular diseases.^44^ To estimate the effects of CaMKII on ER stress and apoptosis in Dox‐induced cardiotoxicity, we measured the degree of ER stress and apoptosis in Dox‐treated hearts treated with KN‐93. Our results demonstrated that KN‐93 markedly inhibited Dox‐induced ER stress and apoptosis by decreasing ER dilation, PERK protein levels and apoptotic rates and elevating GRP78 protein levels. However, 4μ8C, an inhibitor of IRE1α, abrogated the anti‐ER stress and anti‐apoptotic effects of KN‐93, suggesting that IRE1α signalling is involved in the protective effects of CaMKII inhibition against Dox‐induced cardiotoxicity.

IRE1α, a type 1 ER transmembrane protein kinase, initiates a cascade of multiple cellular processes to restore ER homeostasis and promotes cell survival by altering protein synthesis and other cytoprotective signalling in response to stress or protein unfolding and misfolding.^9^, ^45^ XBP1, a transcription factor, is a key regulator of the unfolded protein response signalling pathway in response to ER stress.^46^, ^47^ It has been reported that cardiomyocyte‐specific deletion of XBP1s exacerbates cardiac dysfunction induced by ischaemia/reperfusion, suggesting that XBP1s plays a protective role.^47^ In addition, there is evidence that the ADAMTS13‐TSP1 axis regulates CaMKII activation, which in turn is responsible for heart failure.^25^ However, whether the IRE1α/XBP1s pathway is involved in the beneficial effects of CaMKII inhibition and the underlying mechanisms CaMKII activation in Dox‐induced cardiotoxicity are still to be elucidated. Consistently, our results showed that Dox significantly reduced IRE1α and XBP1s levels. Interestingly, KN‐93 markedly increased IRE1α and XBP1s levels in Dox‐treated mouse hearts. However, inhibiting the IRE1α/XBP1s pathway with 4μ8C significantly reversed the protective effects of CaMKII inhibition against pathological structural damage, cardiac dysfunction, ER stress and apoptosis in Dox‐treated mouse hearts. Moreover, 4μ8C markedly reduced the protein levels of IRE1α and XBP1s induced by CaMKII inhibition in Dox‐treated hearts but had little effect on the levels of CaMKII phosphorylation and PLB phosphorylation. It is important to note that treatment with rhADAMTS13 decreased the protein level of TSP1 and the phosphorylation of CaMKII in Dox‐treated human AC16 cardiomyocytes. These results demonstrate that the ADAMTS13‐TSP1 axis regulates CaMKII activation and exacerbates Dox‐induced cardiotoxicity by triggering endoplasmic reticulum stress and apoptosis by inhibiting the IRE1α/XBP1s pathway.

In summary, the present study provides mechanistic evidence that CaMKII is a potential target for the treatment of Dox‐induced cardiotoxicity that mediates ER stress and apoptosis by regulating the IRE1α/XBP1s pathway. However, considering the functions of CaMKII and relevant substrates, other pathways may also be involved in the protective effects of CaMKII inhibition against Dox‐induced cardiotoxicity. Therefore, further investigation is needed to clarify the precise mechanisms.

## AUTHOR CONTRIBUTIONS

**Lingheng Kong:** Conceptualization (equal); data curation (equal); investigation (equal); methodology (equal); project administration (equal); software (equal); writing – original draft (equal); writing – review and editing (equal). **Yimeng Zhang:** Data curation (equal); formal analysis (equal); investigation (equal); methodology (equal). **Jiayi Ning:** Data curation (equal); formal analysis (equal); investigation (equal); methodology (equal). **Chennian Xu:** Data curation (equal); methodology (equal); software (equal). **Zhenyi Wang:** Methodology (equal). **Jian Yang:** Conceptualization (equal); funding acquisition (equal); project administration (equal); supervision (equal); validation (equal); writing – review and editing (equal). **Lifang Yang:** Conceptualization (equal); funding acquisition (equal); investigation (equal); project administration (equal); resources (equal); supervision (equal); validation (equal); visualization (equal); writing – review and editing (equal).

## FUNDING INFORMATION

This work was supported by grants from the National Natural Science Foundation of China (81774415, 82174493), the Shaanxi Province Fund for Distinguished Young Scholars (2021JC‐49), the Foundation of Xi'an Municipal Bureau of Science and Technology (2019114613YX003SF036) and the Natural Science Basic Research Foundation for the Distinguished Young Scholars of Shaanxi Province (S2018‐JC‐JQ‐0094).

## CONFLICT OF INTEREST

The authors declare no conflicts of interest.

## Supporting information


Figure S1

## Data Availability

The data that support the findings of this study are available on request from the corresponding author.
