# Supplementary figures and images for: CaMKII orchestrates endoplasmic reticulum stress and apoptosis in doxorubicin‐induced cardiotoxicity by regulating the IRE1α/XBP1s pathway
Source: J Cell Mol Med. 2022 Sep 16;26(20):5303–14. doi: 10.1111/jcmm.17560 (PMC9575131; doi:10.1111/jcmm.17560)

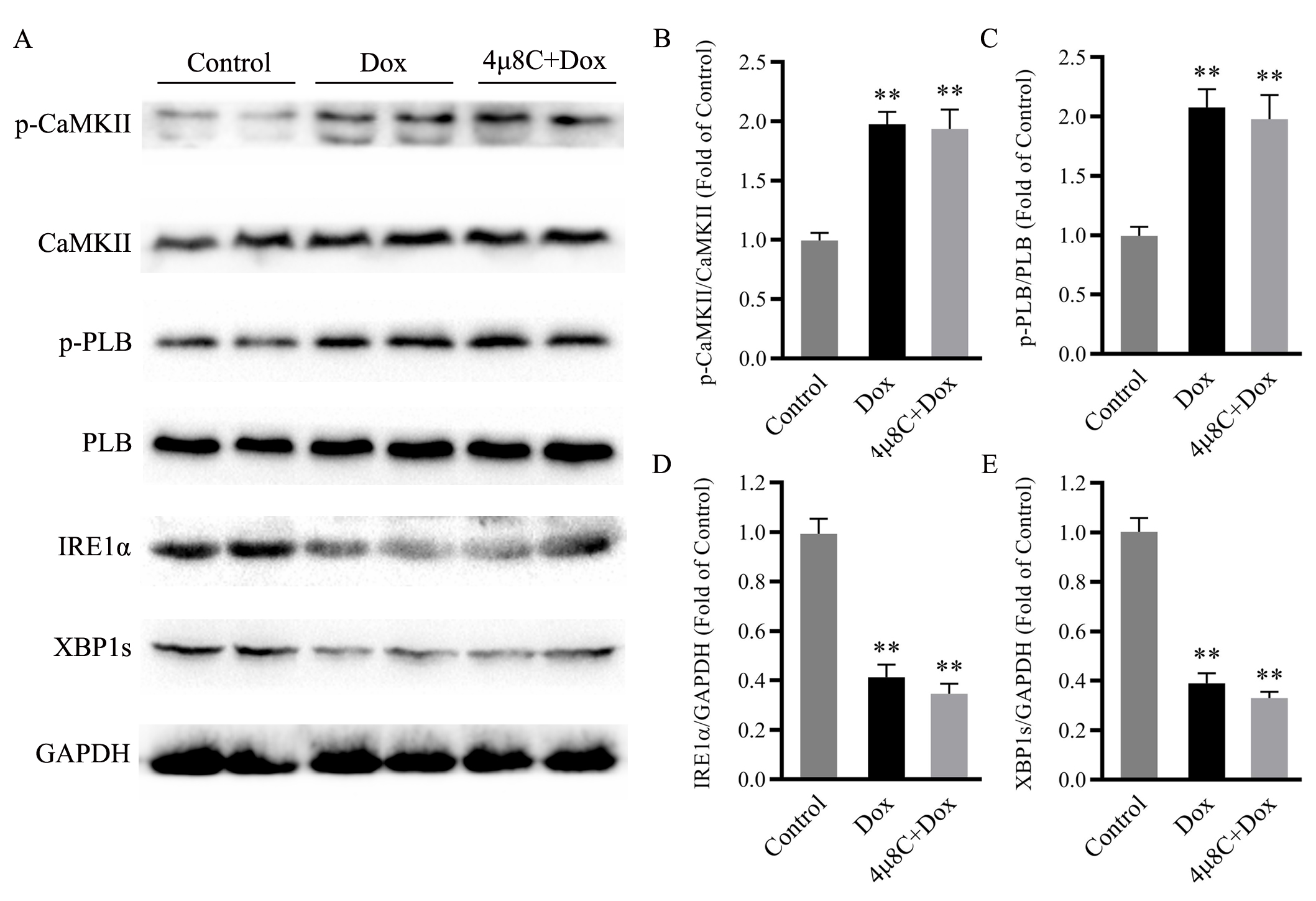

Supplement: Supplementary file 1 — Figure S1 [file JCMM-26-5303-s001.jpg]
